# Fabrication of Dish-Shaped Micro Parts by Laser Indirect Shocking Compound Process

**DOI:** 10.3390/mi7060105

**Published:** 2016-06-20

**Authors:** Huixia Liu, Chaofei Sha, Zongbao Shen, Liyin Li, Shuai Gao, Cong Li, Xianqing Sun, Xiao Wang

**Affiliations:** School of Mechanical Engineering, Jiangsu University, Zhenjiang 212000, China; shachaofei@gmail.com (C.S.); szb@ujs.edu.cn (Z.S.); llyliliyin@163.com (L.L.); gaoshuai2017@163.com (S.G.); Licong183@126.com (C.L.); xianqingyzy@163.com (X.S.); wx@ujs.edu.cn (X.W.)

**Keywords:** laser compound process, dish-shape micro parts, soft punch, deep drawing, punching, blanking

## Abstract

Compound process technology has been investigated for many years on a macro scale, but only a few studies can be found on a micro scale due to the difficulties in tool manufacturing, parts transporting and punch-die alignment. In this paper, a novel technology of combining the laser shock wave and soft punch was introduced to fabricate the dish-shaped micro-parts on copper to solve these difficulties. This compound process includes deep drawing, punching and blanking and these processes can be completed almost at the same time because the duration time of laser is quite short, so the precision of the micro-parts can be ensured. A reasonable laser energy of 1550 mJ made the morphology, depth of deformation, dimensional accuracy and surface roughness achieve their best results when the thickness of the soft punches was 200 μm. In addition, thicker soft punches may hinder the compound process due to the action of unloading waves based on the elastic wave theory. So, the greatest thickness of the soft punches was 200 μm.

## 1. Introduction

The fabrication of miniature parts is becoming more and more important due to the trend of miniaturization and the development of products ranging from mobiles and computers to medical products [1]. Smaller cell phones and wrist watches are needed, so the ability of manufacturing smaller components of these devices is becoming increasingly significant. Some precision instruments contain some micro-mechanical parts such as connector pins, miniature screws, contact springs, chip leadframes and IC (Integrated Circuit) sockets [2]. 

Many processes are involved in the micro-forming method such as stamping, forging, hydro-extrusion, extrusion, bending, superplastic forming, deep drawing, *etc.* [3], but many studies are only limited to a single process. When a compound process is needed to fabricate micro-parts, it becomes hard, especially when the size of the parts is small. Kolleck *et al.* [4] utilized a specific modified die to fabricate the outer shaving foils in electrical shavers with micro-holes with raised edges smaller than Ø 0.53 mm. They summarized the process development and also researched the influence of punch and die geometry on the process forces and the accuracy of parts. Fu *et al.* [5] designed a compound die to fabricate cup-shaped parts within two stages using the material of pure copper. This compound die was used in order to overcome the difficulty in positioning the tiny work piece on the die. A circular copper sheet was first blanked out from the work piece and then drawn into the die cavity to form a cup-shaped micro-part in a single stroke. Flosky *et al.* [6] produced a micro-cup with an outer diameter of 1 mm under the blanking and deep drawing process. The outer diameter punched the work piece into a circular sheet and the inner diameter served as a deep drawing die forming the cup. It shows that the punch edge wears out quicker than the rest part of the tool. Furthermore, it is found that the positioning of the tool has a high influence on the wear behavior. Lee *et al.* [7] developed a miniaturized press system that has a precise compound die which can complete the piercing and blanking process to fabricate thin valve components. The multi-processed experiment of thin foil was carried out and the quality of the fabricated component was good without a burr. 

When it comes to dish-shaped micro-parts involving a compound process as discussed in this article, these types of devices are rather complex, thus increasing the cost of tool manufacturing and prolonging the cycle of manufacturing. In addition, the manufacturing of the punch to fabricate micro-parts is difficult on the micro scale. It should also be noticed that an inappropriate position between the punch and die could cause defect and deformation, broken punches and the injection of the micro-parts [8]. Joo *et al.* [9] developed a micro punching system to make a micro-hole of 100 μm in diameter. The punch was fabricated by a grinding process with a diamond wheel. The stripper was also designed to guide the punch to minimize the possible misalignment and there was a CCD camera to monitor the error between the punch and die. However, for the compound process, this guidance device may become more complex.

With the development of laser machining technology, there is no need to fabricate the punch. Niehoff *et al.* [10] used laser forming technology to make laser deep drawing possible on Al 99.5 with a thickness of 50 μm and discussed the influence of defocusing, the number of pulses, power density, the diameter of the die and the material on the forming properties. Liu *et al.* [11] used the laser-induced shock waves to act as the micro-punch to punch micro-holes of 250 μm in diameter on the sheet metal of 10 μm in thickness by a single pulse, and good edge quality was obtained. In addition, some researchers developed a flexible forming method using rubber or plasticine. Rhim *et al.* [12] used silicone rubbers instead of rigid punches as a pressure transmission medium, and punched arrays of micro-holes on copper and titanium foil successfully. Ramezani *et al.* [13] did experimental and finite element research for a rubber-pad forming process. In the experiment, three types of flexible punches were used and the results showed that the simulation results agree well with the experimental results in terms of the thickness prediction of formed parts. Wang *et al.* [14] used plasticine rather than rubber pads as a soft punch to fabricate micro-channels on the sheet metal. It can be found that the distribution of the array micro-channels and the load force have an influence on the deformation. Liu *et al.* [15] combined a rubber indirect forming method with a laser punching process and finally punched arrays of square holes on Al foils of 20 μm in thickness. Afterwards, Wang *et al.* [16] investigated laser dynamic flexible forming (μLDFF) based on both the simulation and experiment. The results showed that combination of the soft punch and laser beam can increase the depth of deformation compared with the method where the laser beam is directly irradiated on the sheet metal. Moreover, a compound process was also investigated in their further studies. Wang *et al.* [17] used the rubber as a soft punch to fabricate micro-parts with a forming/blanking die on pure copper and titanium sheets and good results were obtained from the experiment. Liu *et al.* [18] combined laser shock waves with rubber to fabricate micro-gears including piercing and blanking based on experimental and simulated research, and discussed process parameters such as laser energy, soft punch properties and blank-holder force on the quality of micro-gears.

This paper aims to combine the laser machining method and the soft punch using silica gel as a pressure transfer medium to complete the laser compound process including deep drawing, punching and blanking. In traditional methods, these kinds of micro-parts mainly have two typical methods of fabrication: compound die and progressive die equipment. However, they all need to fabricate micro-parts step by step to realize the desired part geometry and this may cause errors during the movement of the punch. In addition, they all need to fabricate the punch, and the equipment is always complex, involving multiple stages. In this paper, laser was combined with rubber in order to solve the problem of punch-die alignment because the diameter and position of the laser spot are controllable and easier to adjust instead of designing different sizes of punches. Furthermore, rubber can prevent the laser beam from directly irradiating the work piece, thus obtaining better surface quality. Although it involves multiple processes, it can reduce positioning errors due to the fact that the duration time of the laser is pretty short, on the scale of nanoseconds. So, this type of machining method is ideal for fabricating dish-shaped micro-parts.

## 2. Experiment

### 2.1. Principle of the Laser Indirect Shocking Compound Process

**Figure 1:**
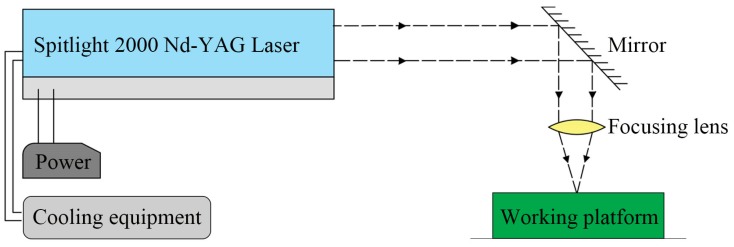
Schematic of the laser machining equipment.

**Figure 2:**
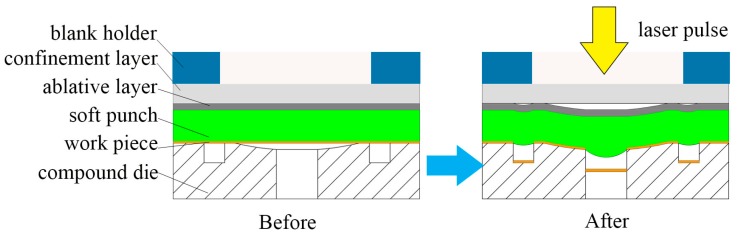
Schematic of the working platform and laser compound process.

Figure 1 shows the laser machining equipment schematically. It consists of power, cooling equipment, a Spitlight 2000 Nd-YAG laser, a mirror, a focusing lens and a working platform. The laser device first irradiates the pulse laser horizontally and then the horizontal laser beam is reflected by a mirror. The mirror is placed at a 45° angle, so the optical path will move downward vertically into the working platform. Figure 2 presents detailed information about the working platform and the laser compound process. The working platform consists of a blanking holder, a confinement layer, an ablative layer, a soft punch, thin sheet metal and a compound die. First, the laser pulse penetrates through the confinement layer. Then laser pulse irradiates on the ablative layer and it vaporizes instantaneously. The vapor absorbs the remaining laser energy and gets ionized into high-temperature and high-pressure plasma [19,20]. The plasma expands quickly and is bounced back by the confinement layer above, thus generating a strong laser shockwave. Under the constraint of the confinement layer and the blanking holder, plasma can only move downward into the soft punch. Under the action of the soft punch and compound die, work pieces are shaped into micro-parts because soft punches are hyper-elastic and incompressible. To conclude, the blanking holder is used to confine the material outside the die cavity. The confinement layer is used to hinder plasmas from expanding upward to magnify the peak pressure and prolong the duration time shockwave pressure. The ablative layer consists of black paint pre-coated on the one side of the silica gel before experiments and it can protect the soft punch from being directly irradiated by the laser pulse and enhance the absorption rate of the laser energy [21]. When one experiment was done, it was necessary to use a new soft punch with an ablative layer because the ablative layer was absorbed by laser beam. The function of the soft punches is to transfer laser energy into mechanical energy acting on the surface of the work piece. 

### 2.2. Experimental Preparation

In this experiment, a Spitlight 2000 Nd-YAG Laser was employed and its main parameters are shown in the Table 1. In order to ensure the accomplishment of the compound process, the diameter of the laser spot should be larger than the maximum diameter of the compound die. Zheng *et al.* [22] suggested that the laser spot diameter should be a little larger than the dimensions of the die in order to get precise micro-parts. The maximum diameter of the compound die is 1.8 mm, so the diameter of the laser spot was set at 2.5 mm. In this case, the diameter of the laser beam is not much larger than the maximum diameter of the compound die, so the distribution of the laser beam cannot be considered as uniform because it follows Gaussian distribution spatially.

**Figure 3:**
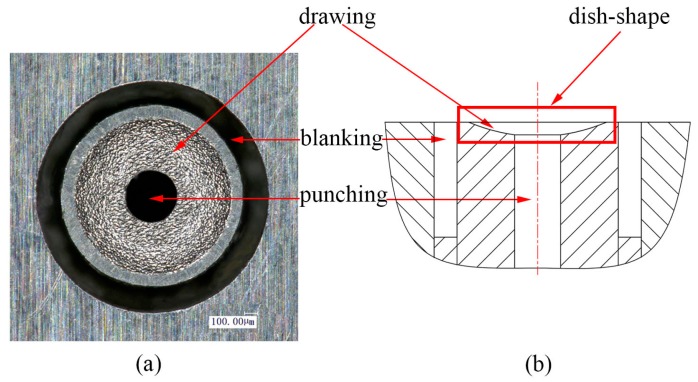
(**a**) Two-dimensional plot of the compound die; (**b**) cross-section of the compound die.

PMMA (Polymethyl Methacrylate) 3 mm in thickness was used as the confinement layer due to the fact that it is transparent and has high mechanical strength. Silica gel was used as the soft punches and the work piece material was pure copper 40 μm in thickness. In order to research the effects of the thickness of the soft punches on the deformation depth, silica gel of 200, 300 and 400 μm in thickness was employed in this article. Both work pieces and soft punches were cut into square pieces of 10 mm × 10 mm and anhydrous alcohol was used to clean the surface of the work pieces before carrying out the experiment. The work piece was placed on the top surface of the compound die. Table 2 shows the thicknesses of the experimental parameters. Figure 3 shows the details of the compound die. Figure 3a shows the two-dimensional (2D) plot of the compound die. An American Iron and Steel Institute (AISI) 1090 mold steel was chosen as the material to fabricate the compound die because of its high stiffness and hardness. The dimensions of the compound die are 50 mm × 50 mm × 5 mm and it was fabricated with micro-electric discharge machining (micro-EDM). This compound die can complete three processes almost at the same time. From Figure 3b, it can be seen that the cross-section of the compound die seems like a dish, so it can be used to fabricate dish-shaped micro-parts.

## 3. Experimental Results and Discussions

### 3.1. The Effect of Laser Energy

**Figure 4:**
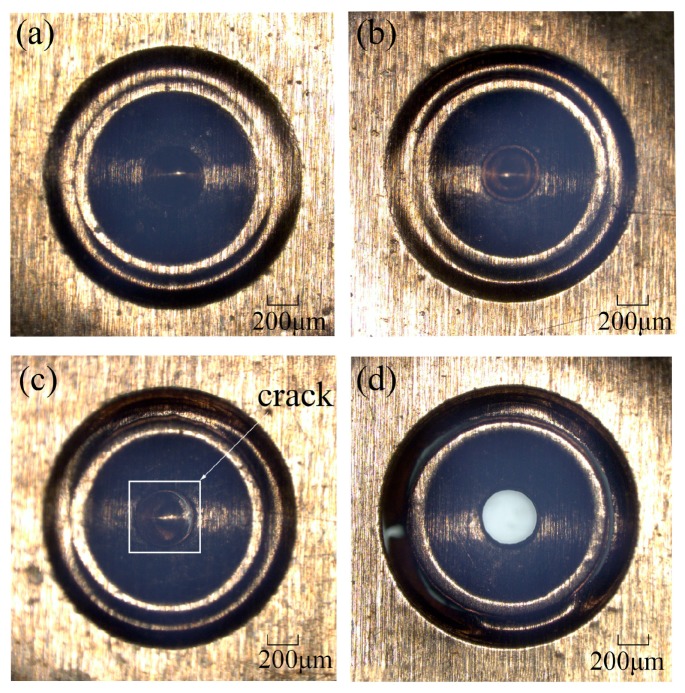
Two-dimensional plot of the work piece when the laser energy is (**a**) 565 mJ; (**b**) 675 mJ; (**c**) 835 mJ; (**d**)1200 mJ.

**Figure 5:**
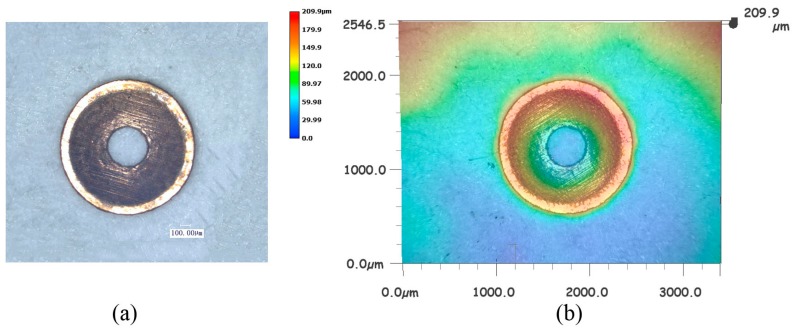
The morphology of a micro-part when a laser energy of 1550 mJ was employed on the work piece: (**a**) 2D plot of the dish-shaped micro-part; (**b**) 3D plot of the dish-shaped micro-part.

Laser energy is one of the key parameters in the process of laser machining, especially in this experiment, and it will affect the morphology of the work piece under different laser energies. Laser energy is not supposed to be too low. If the laser energy is too low, it cannot generate enough laser shock wave pressure to complete the three processes of micro drawing, punching and blanking, and that means it can only achieve partial processes. In order to investigate the effect of laser energy on the compound process, a series of experiments were carried out on the copper foil using the soft punch of 200 μm thickness under different laser energy. The morphology of the work piece was observed by a KEYENCE VHX-1000C digital microscope (by KEYENCE Corporation in Osaka, Japan). As shown in Figure 4a, the process of deep drawing appeared but the hole was not completely punched when the laser energy was 565 mJ. In this period of time, only elastic and plastic deformation happened within the material. Compared with Figure 4a, Figure 4b shows that the deformation of the work piece around the punching hole area became more obvious and severe when more laser energy, 675 mJ, was acted on the material. Furthermore, a crack began to appear when the laser energy was 835 mJ as shown in Figure 4c. Moreover, the punching process finished but the blanking process did not finish under the laser energy of 1200 mJ as shown in Figure 4d. It can be seen that although the inner diameter of the blanking area had almost been cut off from the work piece, a few cracks appeared around the outer diameter of the blanking area. This can be explained by the uneven distribution of the laser energy in space, which means the laser energy in the central area is higher than that in the surrounding area. From this aspect, it is understandable why the punching of the central hole was finished before the blanking of the micro-parts. In addition, the reason why the micro-part cannot be separated from the work piece is because the laser energy is not big enough and the maximum shear stress suffered at the edge of the blanking mold is still less than the dynamic fracture strength of the material itself. Finally, the dish-shaped micro-part was blanked out when the laser energy reached 1550 mJ and Figure 5 shows the positive side of the blanked micro-part. Figure 5a shows the 2D plot of the work piece and Figure 5b shows the three-dimensional (3D) plot. It can be found that the quality of the work piece is fine under the laser indirect shocking compound process. From Figure 4 and Figure 5, it can be seen that the compound process has sequences, namely forming, piercing and blanking with the increase of the laser energy. In traditional methods, the sequence is very important for fabricating this kind of part. For example, if the piercing process is finished first, it could cause some problems because it becomes difficult to ensure the hole’s dimensions when undergoing the forming process. So, the sequence, forming, piercing and blanking can somehow ensure the accuracy.

**Figure 6:**
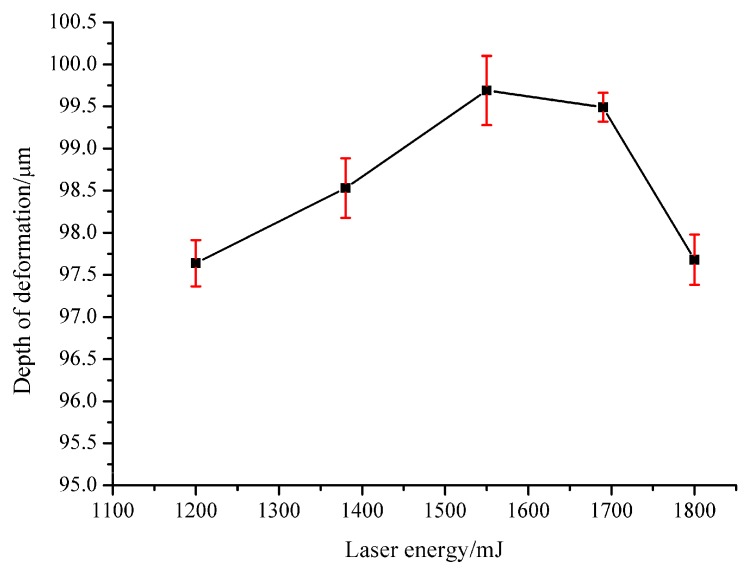
The relationship between the laser energy and deformation depth when the thickness of soft punches was 200 μm.

Although Figure 4 and Figure 5 show the morphology of both work pieces and micro-parts qualitatively, it is still necessary to study the depth of deformation quantitatively because it is an important index by which to evaluate the quality of the part. So, in order to specifically investigate the effect of the laser energy on the compound process, the deformation depth of deep drawing should be considered. In the experiment, laser energies of 1200, 1380, 1550, 1690 and 1800 mJ were used, respectively, when the thickness of the soft punch was 200 μm and that of the work piece was 40 μm. Figure 6 shows the relationship between the laser energy and deformation depth when the thickness of the soft punches was 200 μm. Three experiments were carried out under each laser energy. It can be found that the maximum deformation depth of the work pieces was 99.69 μm (*E* = 1550 mJ) while the deep drawing depth of the compound die was 105 μm. One explanation is that air was enclosed in the compound die cavity and it was compressed gradually during the laser compound process, which led to the resistance force becoming stronger. If the laser energy was too high and the air could not be expelled out, it would prohibit the material flow [23]. So, the deformation depth cannot reach 105 μm. This figure also shows that the deformation depth increased from 97.64 to 99.69 μm with the laser energy when the laser energy was not greater than 1550 mJ. It is mainly because a higher laser energy makes the ablative layer absorb the laser energy more efficiently and more plasmas are generated [19]. When the laser energy increases, more black paint can be vaporized because of the high laser energy. In this case, stronger plasma can be generated. So, generally speaking, it can be seen as a linear process. This means the shock wave pressure produced by the expansion of the plasmas becomes more powerful and larger plastic deformation of the material is achieved in the compound process. In this case, more material could flow into the cavity of the die and the micro-part could attach the rigid mold more closely, so it could increase the depth of deformation. However, it does not mean that more laser energy will lead to better results. When the laser energy is too high, it may lead to a serious thermal effect on the sample [19]. Another issue is that the depth of the deformation decreased when the laser energy was higher than 1550 mJ. It was seen that when the laser energy was 1800 mJ, the deformation depth showed a sharp decrease from 99.49 to 97.68 μm. This can be explained by the fact that when the laser energy is too high, the work piece collides with the compound die fiercely and then generates rebound forces, causing springback of the micro-parts. Therefore, the depth of deformation decreased. Kolleck *et al.* [4] researched the combined micro-forming and punching process and also found the springback behavior of the work piece during the opening stage because of the residual stress on the foil following the punch’s retreat. They also found that when the chamfer angle of the modified blanking die increases, the springback becomes more obvious. Therefore, compared with the single punching process, the combination of punching and forming makes the springback become more severe. The springback is not good for the forming, so it is necessary to choose a proper laser energy to reduce the springback. It can be concluded that the laser energy should be neither too low nor too high and the best laser energy for the maximum deformation depth, 99.69 μm, was at 1550 mJ.

### 3.2. The Dimensional Accuracy of the Punched Holes

**Figure 7:**
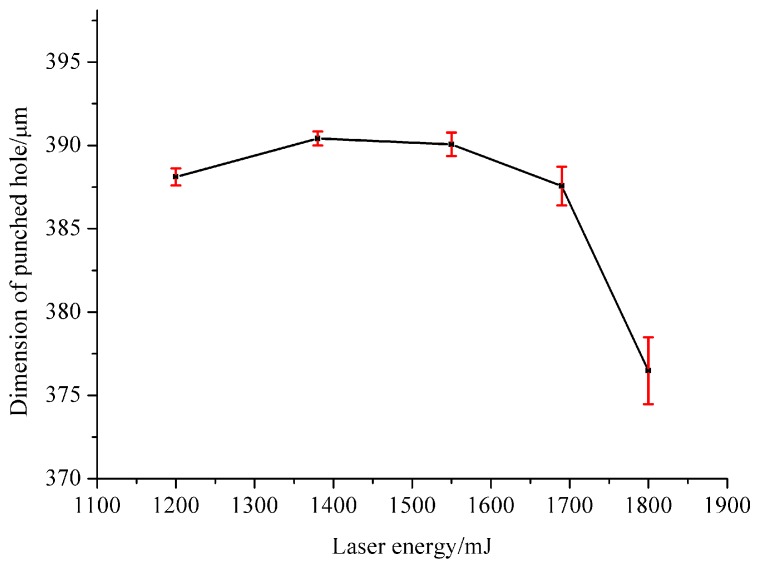
The relationship between laser energy and the dimension of the punched hole.

The punched hole is rather important for the dish-shaped micro-part because this hole can be used to connect with the shaft. In order to study the dimensional accuracy of punched holes, the diameter of the hole was measured under different laser energies. Figure 7 shows the relationship between the laser energy and the dimension of the punched hole and the error bar is the standard deviation. The maximum diameter of the hole was 390.41 μm when the laser energy was 1380 mJ. If the laser energy was 1200 mJ, the diameter was 388.11 μm, lower than that at 1380 mJ. However, when the laser energy was beyond 1380 mJ, the diameter of the hole decreased gradually from 390.41 to 376.48 μm. This indicates that in order to obtain precise micro-parts, a reasonable laser energy should be used and it cannot be too low or too high. It can be seen that when the laser energy reached 1800 mJ, the standard deviation of the diameter value was rather big. It means that higher laser energy may make the diameter value of the micro-parts more unstable. The reason may be that when excessive laser energy was employed, the micro-part stuck to the mold and it was difficult to take it out from the mold, so the dimensional accuracy of the punched hole was poor. On the contrary, when the laser energy was 1380 mJ, the standard deviation of the value was relatively smaller and this means its value was more accurate and stable. So the best laser energy for punching the hole was 1380 mJ and the dimensional accuracy of the hole at 1550 mJ was very close to this value. 

### 3.3. The Effect of the Soft Punch 

Soft punches play important roles in the laser compound process. As the silica gel is hyper-elastic, it is generally assumed to be nearly incompressible during deformation. In traditional methods, an injection device is needed to push parts off the mold and it may make the mold more complicated, especially when the scale of the parts is small. However, the soft punch using silica gel has an adhesive force on the micro-parts and it can stick with the micro-parts, so the micro-part is easier to separate from the compound die. Soft punches can prevent the laser from directly contacting the work piece, so surface quality can be ensured without tool marks [13]. In this study, soft punches with different thicknesses were employed, at 200, 300, 400 μm, respectively. If the thickness of the soft punches is less than 180 μm, the surface quality is poor [16]. In addition, the use of the soft punch can smooth the laser energy deposition [24]. If the soft punch’s thickness is too thin, its ability to homogenize the laser energy is weak and this may cause the work piece to be subjected to non-uniform pressure. That can affect the dimensional accuracy of micro-parts. So, in this case, a thickness under 200 was not employed in the experiment.

**Figure 8:**
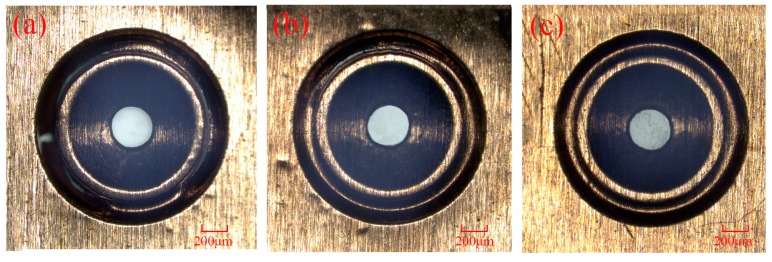
The morphology of the work piece under the laser energy of 1550 mJ when the thickness of the soft punch is (**a**) 200 μm (**b**) 300 μm (**c**) 400 μm.

**Figure 9:**
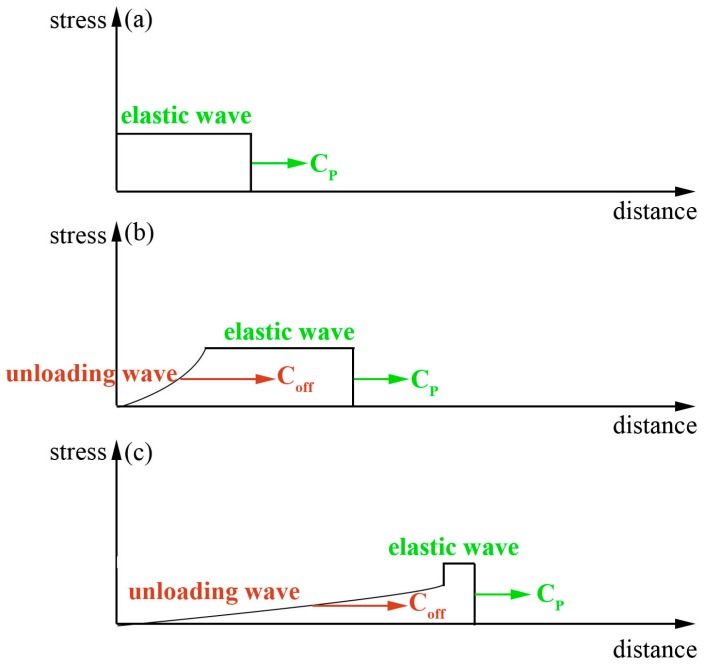
Schematic propagation of elastic waves: *C*_p_ is the velocity of the elastic wave; *C*_off_ is the velocity of the unloading wave. (**a**) Only elastic wave; (**b**) unloading wave occurs; (**c**) the elastic wave is almost be caught up by unloading wave.

Figure 8 shows the effect of the thickness of the soft punches on the compound process under the same laser energy of 1550 mJ. Figure 8b shows that the crack of the work piece becomes smaller than that in Figure 8a when a thicker soft punch of 300 μm was used. In addition, there was no crack when the thickness of the soft punch was 400 μm and the micro-part could not separate from the work piece. When the shock wave induced by the laser acts on the surface of the silica gel, a stress wave appears within the material because of the disturbance that the shock wave applies to the surface of the silica gel. The peak of the stress wave is not beyond the elastic limit of the silica gel because it is very high. So, in this case, the propagation of the stress wave is mainly in the form of an elastic wave and Figure 9 shows the propagation of the elastic wave. At the very beginning, only the elastic wave exists in the process, as shown in Figure 9a. However, when the elastic wave propagates for a period of time, an unloading wave appears and comes to run after the elastic wave, as shown in Figure 9b. In addition, the velocity of the unloading wave is faster than the velocity of the elastic wave. As shown in Figure 9c, the elastic wave is almost chased. So, if the elastic wave traveled for a longer distance, the elastic wave would finally catch up [18,25]. This indicates that when thicker silica gel was used, meaning that the elastic wave tended to travel for a longer distance, the compound process was delayed or hindered in this case.

### 3.4. Surface Roughness

The surface roughness value has a great influence on the dish-shaped micro-parts and it may affect the wear resistance, corrosion resistance and so forth. The destruction of the surface integrity is an important factor for material failure and it is an important mechanical property for the parts. In order to study the surface quality of micro-parts after being impacted by the laser, a high resolution true color confocal microscope (Axio CSM 700, Carl Zeiss, Oberkochen, Germany) was used to measure the surface morphology of micro-parts under laser energies of 1200, 1380, 1550, 1690 and 1800 mJ. 

**Figure 10:**
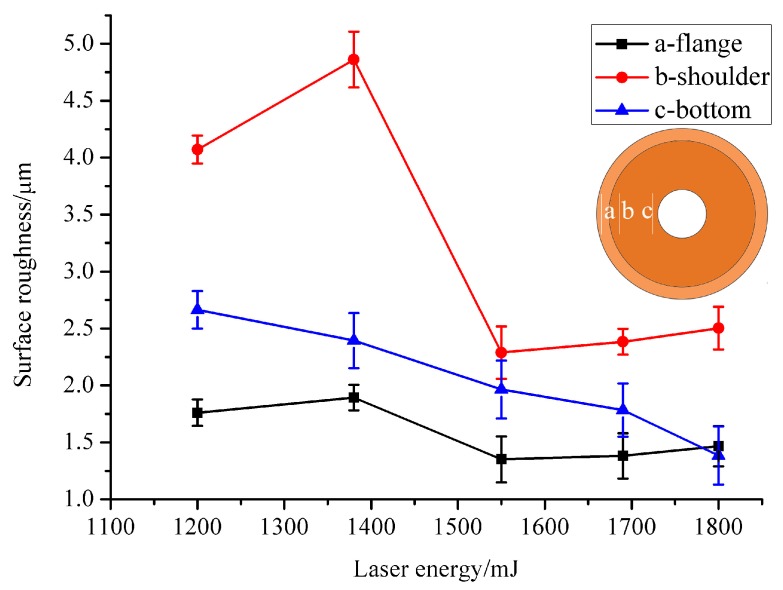
The relationship between the laser energy and the surface roughness value on three different areas.

As shown in Table 3, the surface roughness under different laser energies was measured on three areas, namely the (a) flange, (b) shoulder and (c) bottom. The arithmetical mean deviation (*R*_a_) was used to evaluate the roughness value. Figure 10 shows the relationship between the laser energy and the surface roughness values on three different areas. The surface roughness of three manufactured micro-parts was evaluated under each laser energy. It can be found from Figure 10 that the surface roughness value plunged on the flange and shoulder area when the laser energy reached 1550 mJ. The explanation may be that when the laser energy reached 1550 mJ, the micro-part separated from the work piece completely and, in this case, the micro-part was not restricted by the blank holder anymore. The constraint of the material was reduced and then the contact with the mold became smaller, thus making the surface roughness drop quickly. So, the blanking process has an effect on the surface roughness. In other situations when the laser energy increased from 1200 to 1380 mJ and 1550 to 1800 mJ, the surface roughness value increased with the laser energy. This is because in this experiment, the surface of the deep drawing mold was not grinded and polished in order to ensure its dimensional accuracy, so its surface is a little rough and may cause friction between the material and die. As the laser energy became higher, more plasma was induced and higher laser shock pressure was generated to act on the work piece, leading to a stronger plastic deformation conformal to the underlying three-dimensional rigid mold. Hence, the work piece was more likely to duplicate the features of the mold. However, the bottom area saw a very different trend. As shown in Figure 10, the surface roughness value decreased with the laser energy. This is mainly because the successive laser impact perpendicular to the grinding direction can decrease the peak height of the surface profile induced by grinding [26]. In addition, as the position was closer to the shocked region, the surface quality can be improved [27], which means the surface roughness value decreased. The springback also contributed to the decrease of the surface roughness value. The combined process of forming and punching makes the springback become more obvious as mentioned in Section 3.1. When the springback happened, it could also lead to the decrease of the surface roughness value on the (c) bottom area. So, the combination of forming and punching also has an effect on the surface roughness.

**Figure 11:**
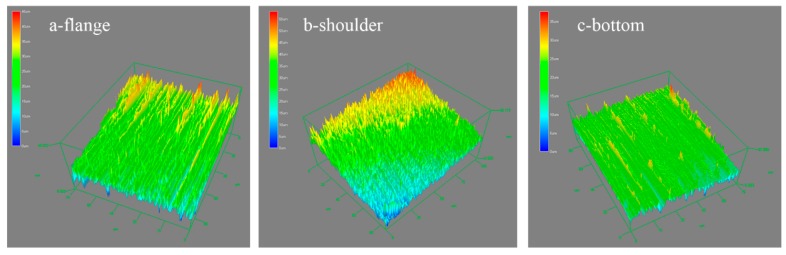
The 3D plot of surface roughness when the laser energy was 1550 mJ.

The surface roughness value is a very important factor for the dish-shaped micro-parts and it is necessary to find a reasonable laser energy to fabricate it. The optimal laser energy for the minimum surface roughness value was 1550 mJ because both the flange, shoulder and bottom area have a relatively good surface quality, as shown in Figure 10 and Figure 11, in the 3D plot of the surface roughness when the laser energy was 1550 mJ.

## 4. Conclusions

In this paper, a dish-shaped micro-part involving deep drawing, punching and blanking was fabricated under the laser shock wave pressure. In the experiment, a novel combination of laser and soft punch was employed to get better results for the dish-shaped micro-parts. The depth of the deformation, the dimensional accuracy of the punched hole, the thickness of the soft punch and the surface quality were investigated. The following results can be obtained:

(1) The laser energy should not be too low; otherwise, only partial processes can be completed because a low laser energy cannot generate enough plasma to reach enough shock wave pressure. When the laser energy was 1200 mJ, a hole could be punched out while the dish-shaped micro-part still could not be blanked out from the work piece. However, if the laser energy reached 1550 mJ, micro-parts could be successfully blanked out from the work piece. The laser energy has a great influence on the depth of the deformation. It can be found that the depth of the deformation increased with the laser energy and the maximum depth was 99.69 μm when the laser energy was 1550 mJ. However, when the laser energy was beyond 1550 mJ, the depth of the deformation decreased due to the springback of the work piece at the bottom area. 

(2) The laser energy can also affect the dimensional accuracy of the punched hole of dish-shaped micro-part. It can be concluded that if the laser energy was 1380 mJ, the best dimensional accuracy could be obtained and the hole’s diameter was 390.41 μm; excessive laser energy can affect the dimensional accuracy of the dish-shaped micro-parts.

(3) The thickness of the soft punch is also an important factor for the fabrication of dish-shaped micro-parts. The morphology of the work piece showed a great difference when different thicknesses of the soft punch were used under the same laser energy. As the soft punch becomes thicker, the unloading wave may catch up to the elastic wave, thus leading to weakness of the shock wave pressure and delaying the laser indirect shocking compound process.

(4) The best laser energy for fabricating the dish-shaped micro-parts was 1550 mJ and it can be found from many different aspects. The minimum laser energy for the micro-part to separate from the work piece was 1550 mJ, as shown in Figure 5. The maximum depth of the deformation could be obtained when the laser energy was 1550 mJ, as shown in Figure 6. The best dimensional accuracy of the punched hole was 1380 mJ, but the dimensional accuracy at 1550 mJ was also good, as shown in Figure 7. The best surface quality was achieved at 1550 mJ.

## Figures and Tables

**Table 1:** Main parameters of Spitlight 2000 Nd-YAG laser.

Parameters	Values
Single pulse energy	80–1800 mJ
Pulse width	8 ns
Wave length	1064 nm
Energy stability	<±1%
Spot diameter	2–5 mm

**Table 2:** The thicknesses of the experimental parameters.

Parameters	Values
PMMA	3 mm
Ablative layer	10 μm
Soft punch	200, 300, 400 μm
Copper	40 μm

**Table 3:** Surface roughness values of three areas under different laser energy.

Laser Energy/mJ	a-Flange(*R*_a_/μm)	b-Shoulder(*R*_a_/μm)	c-Bottom(*R*_a_/μm)
1200	1.761	4.071	2.664
1380	1.894	4.861	2.394
1550	1.351	2.289	1.965
1690	1.382	2.384	1.783
1800	1.467	2.503	1.385
